# Chemerin activates fibroblast-like synoviocytes in patients with rheumatoid arthritis

**DOI:** 10.1186/ar3475

**Published:** 2011-09-29

**Authors:** Kayoko Kaneko, Yoshishige Miyabe, Aiko Takayasu, Shin Fukuda, Chie Miyabe, Masashi Ebisawa, Waka Yokoyama, Kaori Watanabe, Toshio Imai, Kenzo Muramoto, Yuya Terashima, Takahiko Sugihara, Kouji Matsushima, Nobuyuki Miyasaka, Toshihiro Nanki

**Affiliations:** 1 Department of Medicine and Rheumatology, Graduate School of Medical and Dental Sciences, Tokyo Medical and Dental University, 1-5-45, Yushima, Bunkyo-ku, Tokyo, 113-8519, Japan; 2 KAN Research Institute, 3F, Kobe MI R&D Center, 6-7-3, Minatojima-minamimachi, Chuo-ku, Kobe, 650-0047, Japan; 3 Tsukuba Research Laboratories, Eisai Co, Ltd, 5-1-3, Toukoudai, Tsukuba, 300-2635, Japan; 4 Department of Molecular Preventive Medicine, Graduate School of Medicine, University of Tokyo, 7-3-1, Hongo, Bunkyo-ku, Tokyo, 113-0033, Japan; 5 Department of Medicine and Rheumatology, Tokyo Metropolitan Geriatric Hospital, 35-2, Sakae-cho, Itabashi-ku, Tokyo, 173-0015, Japan

## Abstract

**Introduction:**

Chemerin is a chemotactic agonist identified as a ligand for ChemR23 that is expressed on macrophages and dendritic cells (DCs). In this study, we analyzed the expression of chemerin and ChemR23 in the synovium of rheumatoid arthritis (RA) patients and the stimulatory effects of chemerin on fibroblast-like synoviocytes (FLSs) from RA patients.

**Methods:**

Chemerin and ChemR23 expression in the RA synovium was ascertained by immunohistochemistry and Western blot analysis. Chemerin expression on cultured FLSs was analyzed by ELISA. ChemR23 expression on FLSs was determined by immunocytochemistry and Western blot analysis. Cytokine production from FLSs was measured by ELISA. FLS cell motility was evaluated by utilizing a scrape motility assay. We also examined the stimulating effect of chemerin on the phosphorylation of mitogen-activated protein kinase (MAPK), p44/42 mitogen-activated protein kinase (ERK1/2), p38MAPK, c-Jun N-terminal kinase (JNK)1/2 and Akt, as well as on the degradation of regulator of NF-κB (IκBα) in FLSs, by Western blot analysis.

**Results:**

Chemerin was expressed on endothelial cells and synovial lining and sublining cells. ChemR23 was expressed on macrophages, immature DCs and FLSs and a few mature DCs in the RA synovium. Chemerin and ChemR23 were highly expressed in the RA synovium compared with osteoarthritis. Chemerin and ChemR23 were expressed on unstimulated FLSs. TNF-α and IFN-γ upregulated chemerin production. Chemerin enhanced the production of IL-6, chemokine (C-C motif) ligand 2 and matrix metalloproteinase 3 by FLSs, as well as increasing FLS motility. The stimulatory effects of chemerin on FLSs were mediated by activation of ERK1/2, p38MAPK and Akt, but not by JNK1/2. Degradation of IκB in FLSs was not promoted by chemerin stimulation. Inhibition of the ERK1/2, p38MAPK and Akt signaling pathways significantly suppressed chemerin-induced IL-6 production. Moreover, blockade of the p38MAPK and Akt pathways, but not the ERK1/2 pathway, inhibited chemerin-enhanced cell motility.

**Conclusions:**

The interaction of chemerin and ChemR23 may play an important role in the pathogenesis of RA through the activation of FLSs.

## Introduction

Rheumatoid arthritis (RA) is a chronic inflammatory disease characterized by synovial hyperplasia, joint destruction and infiltration of the synovium by immunocytes, including lymphocytes, macrophages and dendritic cells (DCs) [1-3]. In addition to these cells, fibroblast-like synoviocytes (FLSs) play a major role in the pathogenesis of RA [4,5] in that they produce a variety of cytokines, chemokines and matrix-degrading enzymes that mediate interaction with neighboring inflammatory and endothelial cells and are responsible for the progressive inflammation in the joints and destruction of the articular cartilage and bone [6-8].

Chemerin is the ligand protein for ChemR23, a G protein-coupled receptor expressed on macrophages, DCs and natural killer cells (NK cells) [9-11]. Chemerin is characterized as a strong chemoattractant factor for ChemR23-expressing cells and acts at subnanomolar concentrations [9,12]. Chemerin is synthesized as an inactive precursor protein, prochemerin, which binds ChemR23 with low affinity [9,13]. It can be rapidly converted into a full ChemR23 agonist by the proteolytic removal of the last six or seven amino acids by neutrophil-derived proteases (elastase and cathepsin G), mast cell products (tryptase), proteases of the coagulation cascade [14,15] and certain bacterial proteases [16] at the inflammatory site.

Investigators in recent studies have reported that the expression of chemerin correlates with ChemR23-positive cell recruitment in human skin inflammatory diseases, such as systemic lupus erythematosus, oral lichen planus and psoriasis [10,12,17]. Moreover, it has been reported that chemerin and ChemR23 are expressed by human articular chondrocytes [18] and endothelial cells [19,20]. The interaction of chemerin with ChemR23 is assumed to play an important role not only in the migration of macrophages and DCs to the sites of inflammation but also in the mediating inflammatory signaling to articular chondrocytes and endothelial cells. However, there is little information on the expression and function of chemerin in the RA synovium.

In the present study, we analyzed the expression of chemerin and ChemR23 in the RA synovium and evaluated the function of chemerin in cultured FLSs isolated from the synovium of RA patients to explore a possible role of chemerin and ChemR23 interaction in the pathogenesis of RA.

## Materials and methods

### Specimens

Synovial tissue samples were obtained from nine RA patients who fulfilled the American College of Rheumatology diagnostic criteria for RA [21] and from four patients with osteoarthritis (OA) who underwent total knee joint replacement surgery. Signed consent forms were obtained before the operations. All patients with RA and OA were female. In RA patients, the mean age (± SEM) was 71.0 ± 3.7 years, mean disease duration (± SEM) was 10.75 ± 3.5 years, and their mean C-reactive protein concentration (± SEM) was 0.33 ± 0.17 mg/dl. Among the patients with RA, seven patients were seropositive for rheumatoid factor and five were positive for anticitrullinated protein antibodies. Two of the patients with RA were receiving prednisolone, one was being treated with bucillamine (an analogue of D-penicillamine), one was receiving methotrexate monotherapy, one was being treated with methotrexate and etanercept and three were was taking tocilizumab. Three patients were receiving no medication at the surgery. The experimental protocol was approved by the Ethics Committee of Tokyo Medical and Dental University.

### Immunohistochemistry

Immunohistochemical analysis was conducted on OCT compound-embedded sections of frozen synovial tissues. Briefly, 8-μm-thick cryostat sections were fixed in cold acetone (stored at -20°C) for three minutes. Endogenous peroxidase activity was blocked by incubation in 2% H_2_O_2 _in methanol for 15 minutes, and then nonspecific binding was blocked with 10% normal goat serum in PBS for 40 minutes. Serial sections were then incubated for two hours at 4°C with 1 μg/ml rabbit anti-chemerin pAb (affinity-purified from rabbit serum immunized with glutathione *S*-transferase-chemerin (E^21^-S^157^) fusion protein; provided by KAN Research Institute, Kobe, Japan), 1 μg/ml rabbit anti-ChemR23 pAb (Acris Antibodies, Herford, Germany) or normal rabbit immunoglobulin G (IgG) (Sigma-Aldrich, St Louis, MO, USA) as an isotype control. Antibody binding was detected using the EnVision+ Kit (DakoCytomation, Carpinteria, CA, USA).

For immunofluorescence double-staining with CD68, CD1a, DC-LAMP or vimentin, and ChemR23, nonspecific binding was blocked with 1% blocking reagent (Roche, Manheim, Germany) in PBS, and then the sections were incubated overnight at 4°C with rabbit anti-ChemR23 pAb or normal rabbit IgG (control) at 1 μg/ml. The samples were then incubated with Alexa Fluor 568-conjugated goat anti-rabbit IgG (4 μg/ml; Invitrogen, Carlsbad, CA, USA) for one hour at room temperature. Next the sections were incubated overnight with 2.35 μg/ml mouse anti-CD68 mAb (KP1; DakoCytomation), 10 μg/ml mouse anti-CD1a mAb (BL6; Immunotech, Marseille, France), 10 μg/ml mouse anti-DC-LAMP mAb (104.G4; Immunotech) or 1 μg/ml mouse anti-vimentin mAb (V9; DakoCytomation) at 4°C. Subsequently, the samples were incubated with 4 μg/ml Alexa Fluor 488-conjugated goat anti-mouse IgG2a (Invitrogen) or anti-mouse IgG1 (Invitrogen) for one hour at room temperature. The slides were examined using a Biozero Fluorescence Microscope (Keyence, Tokyo, Japan).

For whole-mount staining, human synovial tissues from RA patients were washed vigorously in ice-cold PBS, then fixed and permeabilized with a BD Cytofix/Cytoperm Kit (BD Biosciences, San Jose, CA, USA). The whole-mount specimens of synovial tissues were then stained with rabbit anti-ChemR23 pAb or rabbit IgG as an isotype control, followed by secondary antibodies conjugated with Alexa Fluor 488-conjugated goat anti-rabbit IgG (BD Biosciences). The specimens were further stained with Alexa Fluor 633-labeled phalloidin (BD Biosciences). The specimens were embedded in a 30% solution of glycerol in PBS and analyzed with a DM IRE2 confocal laser scanning microscope (Leica Microsystems, Wetzlar, Germany).

### Cell cultures

Synovial tissues from RA patients were minced and incubated with 0.5 mg/ml collagenase (Sigma-Aldrich) for one hour at 37°C, then pressed through a metal screen to obtain single-cell suspensions. The harvested cells were plated in cell culture plates and incubated with DMEM (Sigma-Aldrich) supplemented with 10% FCS (Sigma-Aldrich). Adherent cells were maintained in the medium as FLSs and used after five passages [22] in the experiments that followed.

For immunofluorescence double-staining of cultured FLSs with ChemR23 and vimentin, FLSs were incubated on eight-well chamber slides (4 × 10^4 ^cells/well) (IWAKI, Tokyo, Japan) at 37°C overnight. The adherent cells were fixed in cold acetone for three minutes. Nonspecific binding was blocked with 10% normal goat serum in PBS, then the cells were incubated overnight at 4°C with rabbit anti-ChemR23 pAb or normal rabbit IgG at 1 μg/ml. Next the cells were incubated with Alexa Fluor 568-conjugated goat anti-rabbit IgG (4 μg/ml) for one hour at room temperature. After that step, the cells were incubated overnight with 1 μg/ml mouse anti-vimentin mAb at 4°C, followed by incubation with 1 μg/ml Alexa Fluor 488-conjugated goat anti-mouse IgG1 for one hour at room temperature. The slides were examined using a Biozero Fluorescence Microscope (Keyence).

### ELISA for chemerin and inflammatory mediators produced by cultured fibroblast-like synoviocytes

FLSs were cultured overnight in 96-well plates (2 × 10^4 ^cells/well) in DMEM with 10% FCS and then incubated with or without recombinant human TNF-α (0.1, 1 or 10 ng/ml) or IFN-γ (1, 10 or 100 ng/ml) (both from R&D Systems, Minneapolis, MN, USA) at 37°C for 48 hours. The concentration of chemerin in culture supernatant was measured with an ELISA kit (R&D Systems) according to the instructions provided by the manufacturer.

To determine the effects of chemerin on the production of IL-6, chemokine (C-C motif) ligand 2 (CCL2) and matrix metalloproteinase 3 (MMP-3) by FLSs, the cells were cultured separately overnight in 96-well plates (4 × 10^4 ^cells/well), then incubated with or without recombinant bioactive human chemerin (2, 10 or 50 nM; R&D Systems) in FCS-free DMEM at 37°C for 24 and 48 hours. The protein levels of IL-6, CCL2 and MMP-3 in the supernatant were assessed using ELISA kits (R&D Systems) according to the manufacturer's instructions. To block the signaling pathway through p44/42 mitogen-activated protein kinase (ERK1/2), p38 mitogen-activated protein kinase (p38MAPK) or Akt, FLSs (4 × 10^4 ^cells/well) were pretreated with specific inhibitors, including a mitogen-activated protein kinase kinase (MEK) inhibitor (10 μM PD98059; Calbiochem, Merck KgaA, Germany), a p38MAPK inhibitor (10 μM SB203580; Calbiochem) or phosphoinositide 3-kinase (PI3K) inhibitor (10 μM LY294002; Cell Signaling Technology, Danvers, MA, USA) for 30 minutes before stimulation with 50 nM chemerin. To confirm the lack of a significant amount of endotoxin in the stimulation procedure, FLSs were incubated with 50 μg/ml polymyxin B in addition to 50 nM chemerin.

### Scrape motility assay

RA FLSs were plated at a density of 1 × 10^5 ^cells/ml in 12-well plates (22.1-mm diameter) in DMEM with 10% FCS. After overnight incubation, the tip of a plastic pipette was drawn across the center of the well to produce a scraped area [23]. The culture wells were washed twice with PBS, and free cells were removed. Then the cells were pretreated with or without pertussis toxin (PTX) (0.5 μg/ml), PD98059 (10 μM), SB203580 (10 μM) or LY294002 (10 μM) for 30 minutes, followed by incubation with 2 or 10 nM of chemerin and CCL2 in FCS-free medium. Immediately after scraping (zero hours) as well as after 24-hour incubation, the process of two-dimensional FLS migration into the cell-free area at the center of the well was photographed with a Nikon TE2000U inverted microscope (Nikon Instruments, Tokyo, Japan) and printed. Cells that migrated into the scraped area after 24 hours were counted by an observer blinded to the study design. The fold increase in the cells was calculated (number of cells with chemerin with or without signal inhibitor per number of cells without treatment).

### Western blot analysis

Synovial tissues from patients with RA and OA were lysed with radioimmunoprecipitation assay (RIPA) buffer (Millipore, Temecula, CA, USA) containing protease inhibitor (Roche) and phosphatase inhibitor cocktail (Sigma-Aldrich) for 30 minutes at 4°C. RA FLSs (3 × 10^5 ^cells/well) were cultured overnight in 60-mm dishes in DMEM with 10% FCS, then the medium was replaced with FCS-free DMEM. This step was followed by incubation with or without recombinant human TNF-α (10 ng/ml), IFN-γ (100 ng/ml), transforming growth factor-β1 (TGF-β1; 1 ng/ml), IL-1β (5 ng/ml) or IL-6 (20 ng/ml) (all from R&D Systems) at 37°C for 24 hours. After incubation, cells were collected and lysed with the RIPA buffer containing protease inhibitor and phosphatase inhibitor cocktail for 30 minutes at 4°C.

A total of 20 μg of protein were boiled in the presence of SDS sample buffer and separated on a 10% SDS-polyacrylamide gel (ATTO, Tokyo, Japan). Proteins were then electrotransferred onto a polyvinylidene fluoride microporous membrane (Millipore) in a semidry system. The membrane was blocked with 5% skim milk for one hour at room temperature, then the immunoblots were incubated overnight with rabbit anti-chemerin pAb (1:1, 000 dilution; Phoenix Pharmaceuticals, Burlingame, CA, USA) or rabbit anti-ChemR23 pAb (1:500 dilution; Abcam, Cambridge, UK) in Can Get Signal Immunoreaction Enhancer Solution (Toyobo Co, Ltd, Tokyo, Japan) at 4°C. Peroxidase-conjugated goat anti-rabbit IgG pAb (GE Healthcare, Birmingham, UK) was used as the secondary antibody.

To detect phosphorylated and total ERK1/2, p38MAPK, c-Jun N-terminal kinase (JNK)1/2 or Akt, cells were incubated in a medium supplemented with 10 nM human recombinant chemerin prior to lysis for 5, 15 or 30 minutes. A total of 7.5 μg of protein was boiled in the presence of SDS sample buffer and separated on 10% SDS-polyacrylamide gel. After blotting, the membranes were blocked with Block Ace blocking solution (Snow Brand Milk Products Co, Ltd, Tokyo, Japan) for one hour at room temperature, then the immunoblots were incubated overnight with rabbit anti-phospho-ERK1/2 pAb (1:1, 000 dilution), rabbit anti-pan-ERK1/2 pAb (1:1, 000 dilution), rabbit anti-phospho-p38MAPK pAb (1:1, 000 dilution), mouse anti-pan-p38MAPK mAb (1:1, 000 dilution) (all from Cell Signaling Technology), mouse anti-phospho-JNK mAb (1:300 dilution; Santa Cruz Biotechnology, Santa Cruz, CA, USA), rabbit anti-pan-JNK pAb (1:1, 000 dilution), rabbit anti-phospho-Akt pAb (1:2, 000 dilution) or rabbit anti-pan-Akt pAb (1:1, 000 dilution) (all from Cell Signaling Technology) in Tris-buffered saline (TBS) containing 0.1% Tween 20 at 4°C. To detect the expression of the regulator of NF-κB, (IκBα), and β-actin, the immunoblots were incubated overnight with rabbit anti-IκBα pAb (1:1, 000 dilution; Santa Cruz Biotechnology) at 4°C or with mouse anti-β-actin mAb (1:5, 000 dilution; Sigma-Aldrich) for one hour at room temperature in TBS containing 0.1% Tween 20. Peroxidase-conjugated goat anti-rabbit IgG pAb (GE Healthcare), rabbit anti-mouse IgG pAb (SouthernBiotech, Birmingham, AL, USA), goat anti-mouse IgG pAb (Promega, Madison, WI, USA) or donkey anti-rabbit IgG pAb (GE Healthcare) was used as the secondary Ab. ECL Plus detection reagent and the ImageQuant LAS 4000 Mini Biomolecular Imager (both from GE Healthcare) were used in conjunction with MultiGauge software (Fujifilm, Tokyo, Japan) to detect and quantitate the bands.

### Statistical analysis

Data are presented as means ± SEM. Student's *t*-tests were applied to compare two groups, and one-way analysis of variance and Dunnett's multiple comparison tests were used to compare three or more groups. *P *< 0.05 was considered statistically significant.

## Results

### Expression of chemerin and ChemR23 in the rheumatoid arthritis synovium

**Figure 1:**
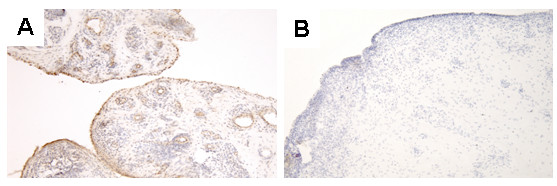
**Expression of chemerin in rheumatoid arthritis and osteoarthritis synovial tissue samples**. Chemerin expression in synovial tissue samples obtained from five RA patients **(A) **and three OA patients **(B) **examined by immunohistochemistry. All sections were counterstained with hematoxylin. OA = osteoarthritis; RA = rheumatoid arthritis.

Strong immunohistochemical staining for chemerin was noted on endothelial cells and synovial lining and sublining cells in the RA synovium (Figure 1A). In contrast, chemerin expression in the OA synovium was minimal (Figure 1B).

**Figure 2:**
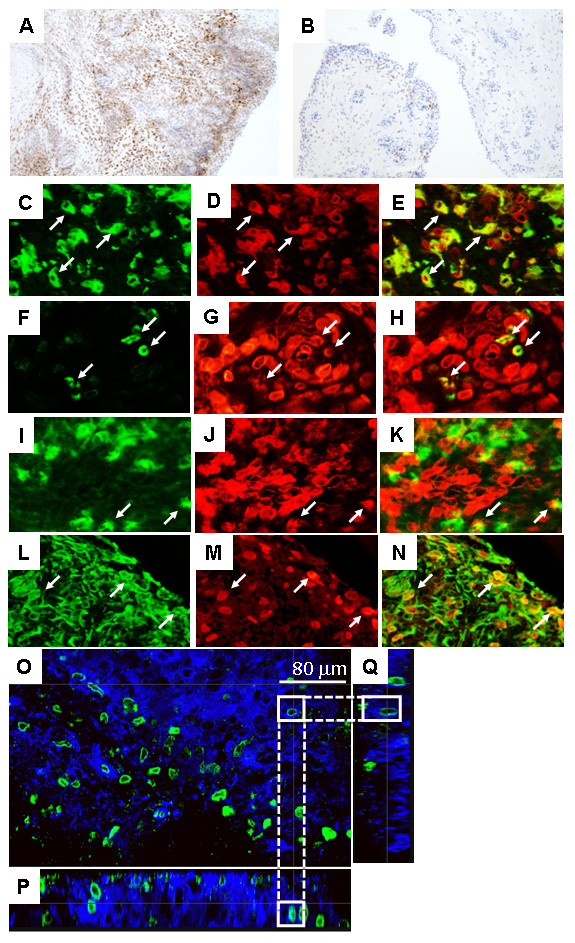
**Expression of ChemR23 in rheumatoid arthritis and osteoarthritis synovial tissue samples**. ChemR23 expression in synovial tissue samples examined by immunohistochemistry that were obtained from five rheumatoid arthritis (RA) patients **(A) **and three osteoarthritis (OA) patients **(B)**. All sections were counterstained with hematoxylin. Sections of RA synovial tissues were also double-stained with CD68, CD1a, DC-LAMP or vimentin, and ChemR23 and were analyzed by fluorescence microscopy as follows: **(C) **CD68, **(D) **ChemR23, **(E) **merged image of (C) and (D), **(F) **CD1a, **(G) **ChemR23, **(H) **merged image of (F) and (G), **(I) **DC-LAMP, **(J) **ChemR23, **(K) **merged image of (I) and (J), **(L) **vimentin, **(M) **ChemR23 and **(N) **merged image of (L) and (M). Arrows indicate double-positive cells. Original magnification ×100 in (A) and (B) and ×400 in (C) through (N). Whole-mount specimens obtained from RA patients were stained with anti-ChemR23 pAb (green) or phalloidin (blue) and then analyzed by confocal laser-scanning microscopy. **(O) ***x*-*y *view of synovial tissue sectioning. *x*-*z *image **(P) **and *y-z *image **(Q) **of synovial tissue sectioning are displayed below and to the right of the *x*-*y *view in (O). Scale bars = 80 μm. White straight line boxes indicate identical cell in each direction (O-Q). Dashed line connects the boxes.

Widespread immunostaining for ChemR23 was noted in all RA samples, with dense staining observed on the sublining cells (Figure 2A). On the other hand, staining in OA samples was much weaker (Figure 2B). Double-staining analysis showed the presence of ChemR23 immunoreactivity on most of the CD68^+ ^macrophages (Figures 2C through 2E), on CD1a^+ ^immature DCs [24,25] (Figures 2F through 2H) and on a few of DC-LAMP^+ ^mature DCs [26] (Figures 2I through 2K). Interestingly, ChemR23 was also expressed on vimentin^+ ^FLSs (Figures 2L through 2N). Furthermore, we performed whole-mount immunostaining of synovial tissues with anti-ChemR23 pAb. The expression of ChemR23 was observed in infiltrated cells in the tissue (Figure 2O). No signal was observed on specimens stained with an isotype-matched IgG control of irrelevant specificity (data not shown). In addition, the *x*-*z *and *y-z *sectioning images obtained by confocal microscopic analysis indicated that ChemR23 was expressed on the surface of the infiltrated cells (Figures 2P and 2Q).

**Figure 3:**
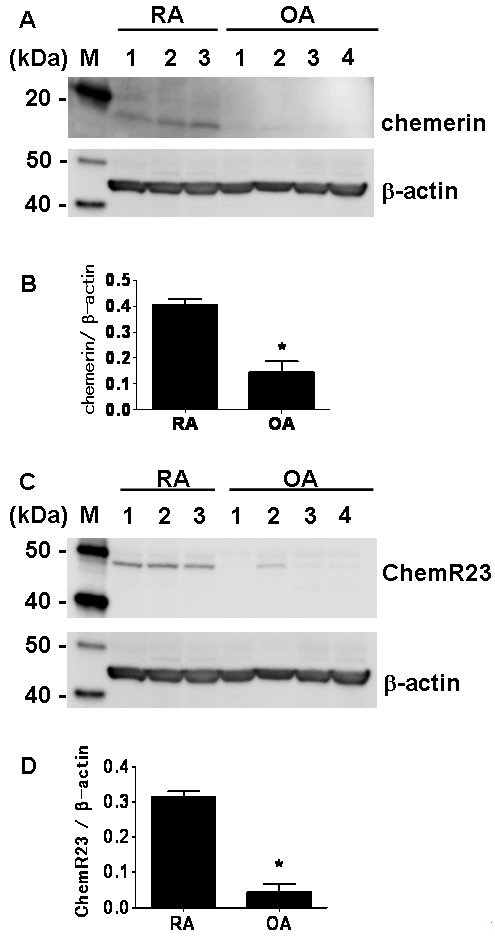
**Expression of chemerin and ChemR23 in rheumatoid arthritis and osteoarthritis synovial tissue samples**. **(A) **Western blots of chemerin protein expression in three rheumatoid arthritis (RA) and four osteoarthritis (OA) synovial tissues. M, protein molecular weight marker. **(B) **Relative protein expression of chemerin to β-actin in RA and OA synovial tissues. *P < 0.05 relative to RA synovial tissue. **(C) **Western blots of ChemR23 protein expression in three RA and four OA synovial tissues. M, protein molecular weight marker. **(D) **Relative expression of ChemR23 protein to β-actin. *P < 0.05 relative to RA synovial tissue.

Next we compared the expression of chemerin and ChemR23 in RA and OA synovial tissues by Western blot analysis. Expression of chemerin by RA synovium was higher than that of OA synovium (Figure 3A). The relative amount of chemerin protein to β-actin in RA was significantly higher than that in OA (Figure 3B). ChemR23 expression by RA synovial tissue was also significantly upregulated compared with OA (Figures 3C and 3D).

### Expression of chemerin and ChemR23 in cultured rheumatoid arthritis fibroblast-like synoviocytes

**Figure 4:**
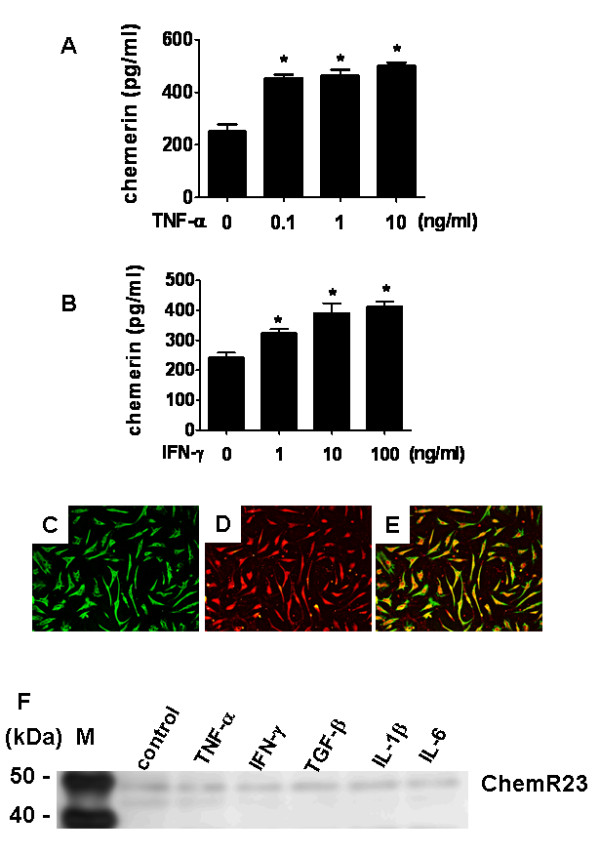
**Expression of chemerin and ChemR23 by rheumatoid arthritis fibroblast-like synoviocytes**. **(A) **and **(B) **Chemerin expression stimulated by TNF-α **(A) **or IFN-γ **(B) **and evaluated by ELISA using supernatants of cultured fibroblast-like synoviocytes (FLSs) isolated from synovial tissue samples taken from rheumatoid arthritis (RA) patients (*n *= 4). FLSs (2 × 10^4 ^cells/well) were stimulated at 37°C for 48 hours with TNF-α (0.1, 1 or 10 ng/ml) (A) or IFN-γ (1, 10 or 100 ng/ml) (A). Data in (A) and (B) are presented as means (± SEM) of one of four independent experiments analyzed in triplicate. *P < 0.05 relative to control. **(C) **through **(E) **Immunocytochemistry showing double-staining of cultured RA FLSs with **(C) **vimentin **(D) **ChemR23 and **(E) **a merged image of (C) and (D). **(F) **Western blot of ChemR23 protein expression in RA FLSs following TNF-α (10 ng/ml), IFN-γ (100 ng/ml), transforming growth factor (TGF)-β1 (1 ng/ml), IL-1β (5 ng/ml) or IL-6 (20 ng/ml) stimulation at 37°C for 24 hours.

The expression of chemerin on cultured FLSs isolated from the RA synovium was analyzed by ELISA. Chemerin was produced by unstimulated FLSs, and the production was significantly upregulated by stimulation with TNF-α and IFN-γ (*P *< 0.05) (Figures 4A and 4B). IL-1β, IL-6 and TGF-β1 did not show any effect on chemerin production (data not shown).

ChemR23 expression on FLSs was determined by immunocytochemical analysis and Western blot analysis. Double-staining revealed that cultured FLSs expressed both ChemR23 and vimentin (Figures 4C through 4E). The use of a specific ChemR23 Ab also showed its expression in RA FLSs on Western blots (Figure 4F). Stimulation with TNF-α, IFN-γ, TGF-β, IL-1β and IL-6 did not show any effect on the expression of ChemR23 in RA FLSs.

### Chemerin enhances IL-6, CCL2 and MMP-3 production by fibroblast-like synoviocytes

**Figure 5:**
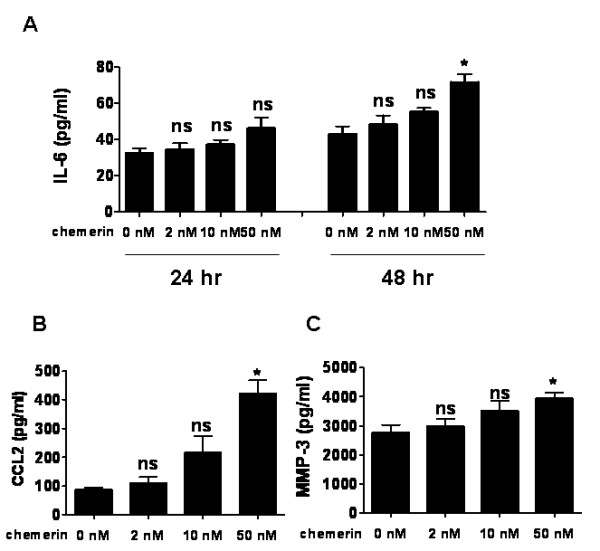
**Chemerin enhances IL-6, chemokine (C-C motif) ligand 2 and matrix metalloproteinase 3 production by rheumatoid arthritis fibroblast-like synoviocytes**. IL-6 **(A)**, chemokine (C-C motif) ligand 2 (CCL2) **(B) **and matrix metalloproteinase 3 (MMP-3) **(C) **expression levels were measured by ELISA using supernatants of cultured fibroblast-like synoviocytes isolated from the synovium of four patients with rheumatoid arthritis. FLSs (4 × 10^4 ^cells/well) were stimulated with 2, 10 or 50 nM recombinant chemerin at 37°C for 24 and 48 hours. Data are presented as means (± SEM) of one of four independent experiments analyzed in quadruplicate. *P < 0.05 relative to samples not treated with chemerin. ns = not significant.

We next evaluated the effects of chemerin on the production of inflammatory mediators by RA FLSs. The cells were stimulated with chemerin for 24 and 48 hours, and the concentrations of IL-6 in the culture supernatant were measured by ELISA. After stimulation with chemerin for 24 hours, IL-6 production from FLSs was moderately increased, although it was not statistically significant. The incubation for 48 hours showed significant upregulation of chemerin-induced IL-6 production from FLSs (*P <*0.05) (Figure 5A). The expression of CCL2 and MMP-3 from FLSs was also enhanced by incubation with chemerin for 48 hours in a dose-dependent manner (*P <*0.05) (Figures 5B and 5C).

### Chemerin enhances cell motility of rheumatoid arthritis fibroblast-like synoviocytes

**Figure 6:**
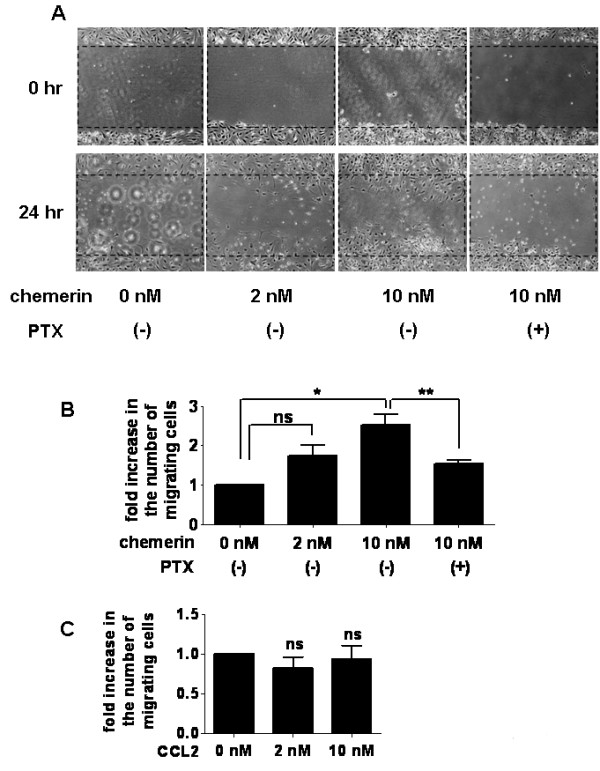
**Chemerin promotes cell motility of rheumatoid arthritis fibroblast-like synoviocytes**. **(A) **A scraped cell-free area was created on a monolayer of fibroblast-like synoviocytes (FLSs). After incubation with serum-free DMEM containing chemerin (2 or 10 nM) with or without pertussis toxin (PTX) (0.5 μg/ml), the process of two-dimensional FLS migration into the cell-free area at the center of the well was photographed with an inverted microscope at 0 and 24 hours. Representative photographs of three separate experiments are shown **(A)**. The number of the cells in the scraped area was counted. Data are fold increases in the number of migrating cells at 24 hours (number of cells that migrated after chemerin stimulation compared to the number of cells without stimulation) **(B)**. Data are presented as means (± SEM) of one of three independent experiments analyzed in triplicate. *P < 0.05 relative to samples not treated with chemerin. **P < 0.05 relative to samples not treated with PTX; ns, not significant. **(C) **Fold increase in the number of migrating cells when FLS were stimulated with 2 or 10 nM of chemokine (C-C motif) ligand 2 (CCL2) for 24 hours is shown; ns = not significant.

In the RA synovium, the migration of RA FLSs into the cartilage and bone is considered important for pannus development [27]. Therefore, by using a scrape motility assay, we investigated whether chemerin could directly alter the migratory behavior of these cells. As shown in Figures 6A and 6B, exogenously added chemerin significantly increased the number of cells that migrated to the scraped area (*P *< 0.05). Moreover, incubation with PTX significantly suppressed chemerin-induced FLS motility (Figures 6A and 6B). Since PTX was previously reported to inhibit signal transduction in ChemR23^+ ^cells by ribosylation of the α_i _subunits of the heterotrimeric G protein of ChemR23 [9], our result suggested the involvement of ChemR23 with chemerin-induced FLS motility. We also evaluated the effect of CCL2 on FLS migration. Incubation with CCL2 (2 or 10 nM) did not promote cell motility of FLSs (Figure 6C).

### Chemerin induces activation of ERK1/2, p38MAPK and Akt of fibroblast-like synoviocytes

**Figure 7:**
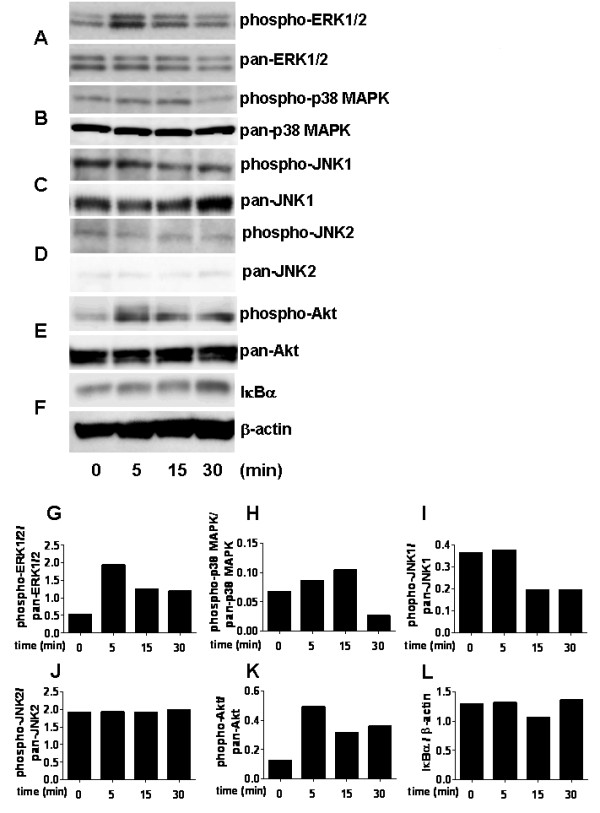
**Chemerin activates p44/42 mitogen-activated protein kinase, p38 mitogen-activated protein kinase and Akt in rheumatoid arthritis fibroblast-like synoviocytes**. Rheumatoid arthritis (RA) fibroblast-like synoviocytes (FLSs) were left unstimulated (zero minutes) or stimulated with 10 nM chemerin for 5 to 30 minutes. Cell lysates were examined by Western blot analysis with anti-phospho-p44/42 mitogen-activated protein kinase (anti-phospho-ERK1/2) and pan-ERK1/2 **(A)**, phospho-p38 mitogen-activated protein kinase (phospho-p38MAPK) and pan-p38MAPK **(B)**, phospho-c-Jun N-terminal kinase (pan-JNK)1/2 and pan-c-pan-JNK1/2 **(C) **and **(D)**, phospho-Akt and pan-Akt **(E) **and regulator of NF-κB (IκBα), and β-actin **(F)**. Shown are band intensity (expressed in arbitrary units) of phospho-ERK1/2 **(G)**, p38MAPK **(H)**, JNK1 **(I)**, JNK2 **(J) **and Akt **(K) **normalized to pan-ERK1/2, p38MAPK, JNK1, JNK2 and Akt, respectively. Relative expression of IκBα protein to β-actin is also shown **(L)**. The results are representative of three different experiments performed using RA FLSs from three different patients.

To determine the signaling pathway of chemerin-induced stimulation of FLSs, we stimulated FLSs with 10 nM chemerin for various time periods and performed Western blot analysis with phospho-specific Abs against ERK1/2, p38MAPK, JNK1/2 and Akt. Unstimulated FLSs showed only minimal phosphorylation of ERK1/2 (at zero minutes), whereas a marked increase in phosphorylation was detected after five minutes of stimulation with chemerin (Figures 7A and 7G). Cell lysates were also examined for chemerin-induced phosphorylation of other MAP kinases, including p38MAPK and JNK1/2. The addition of chemerin augmented the phosphorylation of p38MAPK at 5 to 15 minutes compared with unstimulated RA FLSs, but the level decreased at 30 minutes (Figures 7B and 7H), whereas phosphorylation of JNK1/2 was not promoted by chemerin stimulation (Figures 7C, D, I and 7J). Next, Akt activation, which is linked to regulation of proinflammatory cytokine production by RA FLSs [28], was examined. Figures 7E and 7K show that stimulation of RA FLSs with 10 nM chemerin resulted in enhanced phosphorylation of Akt, with a peak level occurring at five minutes.

IκBα is one of the regulatory proteins of NF-κB, a transcriptional factor which induces various proinflammatory cytokines, including TNF-α and IL-6 [29,30]. When the cells were stimulated for NF-κB activation, IκBα was degraded and the amount of the protein was decreased [31]. Therefore, we analyzed the effect of chemerin on the degradation of IκBα in FLSs to clarify whether NF-κB is involved in chemerin-induced FLS activation. Figures 7F and 7L indicate that chemerin does not induce IκBα degradation in FLSs.

### Involvement of ERK1/2, p38MAPK and Akt pathways in chemerin-induced IL-6 production and cell motility of fibroblast-like synoviocytes

**Figure 8:**
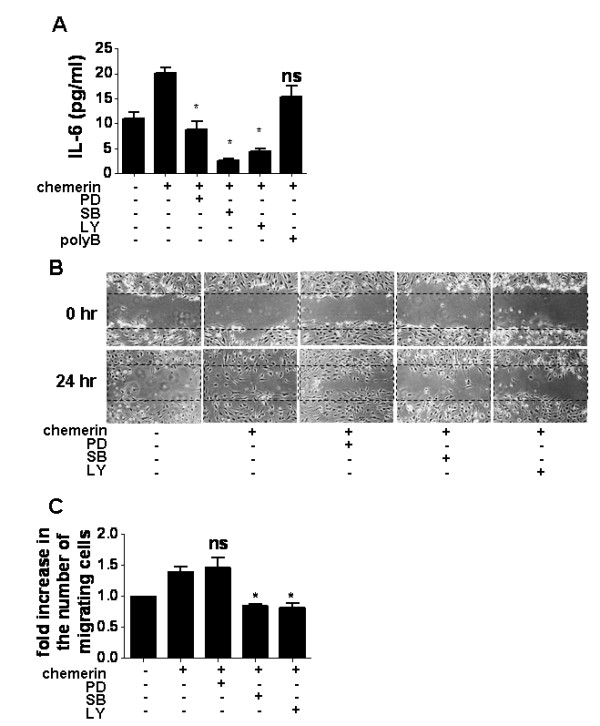
**Involvement of mitogen-activated protein kinase and phosphoinositide 3-kinase-Akt pathways in chemerin-induced fibroblast-like synoviocyte activation**. **(A) **Rheumatoid arthritis (RA) fibroblast-like synoviocytes (FLSs) were pretreated for 30 minutes with PD98059 (mitogen-activated protein kinase kinase inhibitor, 10 μM), SB203580 (p38 mitogen-activated protein kinase inhibitor, 10 μM), LY294002 (phosphoinositide 3-kinase inhibitor, 10 μM), or polymyxin B (50 μg/ml) before stimulation with or without 50 nM chemerin for 48 hours. IL-6 production in the supernatant was measured by ELISA. Data are presented as means ± SEM of one of four independent experiments analyzed in quadruplicate. LY = LY294002; PD = PD98059; polyB = polymyxin B; SB = SB203580. *P < 0.05 relative to treatment with 50 nM chemerin; ns, not significant. **(B) **and **(C) **Effects of PD98059, SB203580 and LY294002 on FLS motility were assessed by scrape motility assay. Representative two-dimensional photographs of FLS migratory process into the scraped cell-free area (0 and 24 hours) are shown in (B). The number of migrating cells in the scraped area was counted. Data represent the fold increase in the number of migrating cells, shown in (C). Data are presented as means ± SEM of one of four independent experiments analyzed in triplicate. *P < 0.05 relative to samples not treated with inhibitor; ns, not significant.

Next we examined the effects of signal blockade on IL-6 production and cell motility of FLSs to evaluate the involvement of signal transduction in chemerin-induced FLS activation. PD98059 (MEK, upstream of ERK1/2, inhibitor [32]), SB203580 (p38MAPK inhibitor [33]) and LY294002 (PI3K, upstream of Akt, inhibitor [34]) were used to block each signaling pathway. Pretreatment with 10 μM PD98059, SB203580 and LY294002 significantly inhibited chemerin-induced IL-6 production by RA FLSs (*P *< 0.05) (Figure 8A). The chemerin-induced enhancement of IL-6 production was not inhibited by polymyxin B, suggesting the lack of a significant amount of endotoxin in this procedure. Furthermore, chemerin-enhanced cell motility was inhibited by SB203580 and LY294002, but not by PD98059 (Figures 8B and 8C).

## Discussion

In this study, we have shown that chemerin and ChemR23 are highly expressed in RA synovium. Chemerin enhanced the production of IL-6, CCL2 and MMP-3 from RA FLSs and promoted cell motility. These results suggest that chemerin activates FLSs in the RA synovium and is probably involved in the pathogenesis of RA.

Our results show upregulation of chemerin and accumulation of ChemR23^+ ^cells in RA synovium. We found that chemerin was expressed on synovial lining and sublining cells and on endothelial cells. Moreover, our study shows that FLSs isolated from RA synovium also produce chemerin *in vitro *and that this production is upregulated by stimulation with TNF-α and IFN-γ, which characterize the inflammatory environment in the RA synovium. In this regard, it was reported that the concentration of chemerin in the synovial fluid was much higher in RA than that in OA (358 ng/ml vs < 1 ng/ml) [9]. ChemR23 was expressed on most macrophages, CD1a^+ ^immature DCs and FLSs in RA synovium. A few DC-LAMP^+ ^mature DCs also expressed ChemR23. It was previously reported that ChemR23 was expressed on immature DCs derived from peripheral blood and that the expression was downregulated by maturation induced by lipopolysaccharide or CD40L [9].

The unique pathway of activation of the chemerin precursor, prochemerin, has been well-investigated [13-15,35,36]. Prochemerin is considered to be activated through C-terminal six- or seven-amino acid processing by protease and to be produced by neutrophils and mast cells at inflammatory sites. Neutrophils and mast cells are known to release protease-rich granules and to secrete immune mediators to activate themselves and other immune cells, triggering positive regulatory feedback that leads to acute or chronic RA inflammation [37-39]. Considered together, our data suggest that chemerin is produced at high levels by FLSs in RA synovium and that extracellular protease is produced by preexistent neutrophils and mast cells converted from inactive prochemerin into bioactive chemerin, which have powerful chemoattractant properties for migration of macrophages and DCs into the RA synovium.

The present results reveal that the expression of ChemR23 on RA FLSs and chemerin itself activate FLSs to enhance the production of IL-6 and CCL2. IL-6 is considered to have pleiotropic functions, including the regulation of maturation and activation of T and B cells, macrophages, osteoclasts, chondrocytes and endothelial cells in RA [40,41]. Clinically, tocilizumab, a humanized mAb specific for IL-6R, has outstanding anti-inflammatory effects, including suppression of disease activity and erosive progression in patients with RA that is resistant to traditional disease-modifying antirheumatic drugs [42]. CCL2 is known as a prototype chemokine that attracts monocytes, T cells, NK cells and basophils into the RA synovium [43]. Therefore, our results indicate that chemerin may be involved in the enhancement of local proinflammatory cytokine and chemokine production by RA FLSs, leading to persistent amplification of inflammation in the RA synovium, possibly in an autocrine or paracrine manner.

The present results indicate that chemerin enhances the cell motility of RA FLSs, whereas CCL2 does not promote FLS migration by this examination procedure. These data suggest that the effect of chemerin for FLS mobility is not affected by inducing the production of CCL2. In the RA joints, the pannus tissue migrates and invades the cartilage and bone, which contribute to damaging these structures [44]. FLSs are the predominant cell type in pannus tissue, especially at the pannus-cartilage junction [45]. FLSs retrieved from synovial tissues directly cause cartilage degradation when cocultured with macrophages *in vitro *[46], suggesting that FLS migration and invasion play a central role in pannus tissue-related cartilage degradation in RA. Moreover, our results show that chemerin enhances MMP-3 production from RA FLSs, which is a proteolytic enzyme with cartilage degradation properties. Collectively, our results suggest that chemerin plays an important role in cartilage destruction through FLS activation.

The present results show that chemerin enhances the activation of ERK1/2, p38MAPK and Akt, but not of JNK1/2 and NF-κB, in FLSs. Moreover, pretreatment with a specific inhibitor of MEK, p38MAPK, and PI3K suppressed chemerin-induced IL-6 production, and p38 MAPK and PI3 kinase inhibitor reduced RA FLS cell motility. These results suggest the involvement of both the MAPK (including MEK-ERK1/2 and p38MAPK) and PI3K-Akt pathways in chemerin-induced IL-6 production by RA FLSs. The p38MAPK and PI3K-Akt pathways are also involved in cell motility induced by chemerin. Chemerin activated macrophage adhesion to fibronectin by activation of p38MAPK and PI3K-Akt signaling pathway [47]. These results suggest that chemerin activates macrophages as well as FLSs in RA synovium.

## Conclusions

Our results identify the important role of chemerin in the activation of FLSs in RA synovium, suggesting that chemerin and ChemR23 interaction might play a role in the pathogenesis of RA.

## Abbreviations

CCL2: chemokine (C-C motif) ligand 2; DC: dendritic cell; DMEM: Dulbecco's modified Eagle's medium; ELISA: enzyme-linked immunosorbent assay; ERK1/2: p44/42 mitogen-activated protein kinase; FCS: fetal calf serum; FLS: fibroblast-like synoviocyte; IFN: interferon; IL: interleukin; JNK: c-Jun N-terminal kinase; mAb: monoclonal antibody; MAPK: mitogen-activated protein kinase; MEK: mitogen-activated protein kinase kinase; MMP: matrix metalloproteinase; NF-κB: nuclear factor κB; NK cells: natural killer cells; OA: osteoarthritis; pAb: polyclonal antibody; PBS: phosphate-buffered saline; PI3K: phosphoinositide 3-kinase; PTX: pertussis toxin; RA: rheumatoid arthritis; TBS: Tris-buffered saline; TGF-β1: transforming growth factor β1; TNF-α: tumor necrosis factor α.

## Competing interests

KMu is an employee of Eisai Co, Ltd. The authors declare that they have no competing interests.

## Authors' contributions

KK participated in the design of the study, carried out the experiments and statistical analysis and drafted the manuscript. YM, AT, SF, CM, WY, ME and KW assisted with carrying out the experiments and collecting data. TI, KMu, YT and KMa assisted in data interpretation and manuscript preparation. TS collected the clinical material. TN and NM conceived the study, participated in its design and coordination and helped to draft the manuscript. All authors read and approved the final manuscript for publication.
